# Validation study of the Korean version of the parent protection scale

**DOI:** 10.1186/s41687-026-01170-6

**Published:** 2026-08-05

**Authors:** Ji Yoon Baek, Jee Na Kim, Yubin Park, Yerin Yhee, Jin-ah Sim, Aesun Shin, Jung Min Ko

**Affiliations:** 1 Department of Preventive Medicine, Seoul National University College of Medicine, Seoul, Korea, Republic of; 2 Integrated Major in Innovative Medical Science, Seoul National University Graduate School, Seoul, Korea, Republic of; 3 Department of Nursing, Seoul National University College of Nursing, Seoul, Korea, Republic of; 4 Division of Clinical Genetics, Department of Pediatrics, Seoul National University College of Medicine, Seoul, Korea, Republic of; 5 School of Business Administration, American University of Sharjah, Sharjah city, United Arab Emirates; 6 Graduate School of Public Health and Healthcare Management, The Catholic University of Korea, Seoul, Korea, Republic of; 7 Population and Quantitative Health Sciences, UMass Chan Medical School, Massachusetts Worcester, USA; 8 Cancer Research Institute, Seoul National University, Seoul, Korea, Republic of; 9 Interdisciplinary Program in Cancer Biology Major, Seoul National University College of Medicine, Seoul, Korea, Republic of; 10 Rare Disease Center, Seoul National University Hospital, Seoul, Korea, Republic of

**Keywords:** Validation study, Reliability, Validity, Pediatrics, Cancer, Rare disease

## Abstract

**Background:**

Protective parental behavior is crucial for children’s safety and emotional well-being, but excessive protection may hinder their autonomy. This issue is particularly relevant for caregivers of children with chronic or rare diseases, who may adopt heightened protective behaviors. The Parent Protection Scale (PPS) is a validated patient-reported tool for assessing parental overprotection. Given Korea’s unique cultural context, which emphasizes close family ties, this study aimed to translate and validate PPS for Korean populations.

**Methodology:**

A total of 179 caregivers of pediatric patients aged 5–10 years with cancer or rare diseases were recruited from eight hospitals across Korea between August 2023 and December 2024. The PPS was translated into Korean through a forward-backward translation process. Exploratory factor analysis (EFA) with principal axis factoring and direct oblimin rotation was performed to examine the factor structure. Internal consistency was assessed by Cronbach’s alpha, and concurrent validity was evaluated using Pearson correlation with questionnaires for anxiety, depression, and social support measures.

**Results:**

EFA revealed a four-factor structure, which differed from that of the original PPS subscales—strict behavioral control, emotional intrusiveness, anxious ambivalence, and autonomy restriction—accounting for 44.85% of the total variance. Internal consistency was acceptable for the total scale (α = 0.79) and moderate for two subscales (anxious ambivalence and autonomy restriction; α = 0.66–0.75) but lower for the others (strict behavioral control and emotional intrusiveness; α = 0.57–0.64). Significant negative correlations were found between strict behavioral control, anxious ambivalence, and autonomy restriction and caregiver anxiety, depression, and social support, supporting concurrent validity. No significant associations were observed between emotional intrusiveness and caregiver anxiety, depression, or social support.

**Conclusions:**

This study provides evidence that the Korean PPS is a culturally adapted tool with acceptable overall reliability for assessing parental protection, with the potential to inform family-centered care and culturally adapted interventions. In clinical practice, it may support more precise identification of protective parenting patterns and guide targeted interventions to balance protection with the promotion of child autonomy.

**Supplementary Information:**

The online version contains supplementary material available at https://doi.org/10.1186/s41687-026-01170-6.

## Background

Protective parental behavior plays a vital role in caregiving by ensuring children’s physical safety and emotional well-being [1]. Although such behaviors are generally adaptive, excessive parental protection may restrict opportunities for children to develop autonomy, independence, and age-appropriate coping skills, and may be associated with adverse psychosocial outcomes [2–4]. This issue may be particularly relevant among caregivers of children with chronic or rare diseases, for whom ongoing health concerns, uncertainty, and caregiving demands may contribute to heightened protective parenting behaviors [5, 6].

Levy originally conceptualized parental overprotection as a pattern of parenting characterized by excessive parent‒child contact, prolonged infantilization, restriction of independent behavior, and excessive parental control [7]. Subsequent research and parent‒child relationship measures further refined the conceptualization of parental overprotection. Notable examples include the Parent‒Child Relations Questionnaire, which captured restrictive and controlling parental behaviors, the Parental Bonding Instrument, which assessed perceived parental care and protection, and the Mother-Child Relationship Evaluation, which differentiated overprotective from overindulgent parenting styles [4, 8, 9]. Together, these theoretical and empirical perspectives established parental overprotection as a multidimensional construct and provided a conceptual foundation for the development of the Parent Protection Scale (PPS) [4].

Based on this conceptual framework, the PPS was developed for caregivers of children aged 2 to 10 years to assess parental overprotection across four key subscales: supervision, separation difficulties, dependence, and control [4, 10]. It was originally designed to quantify caregivers’ protective and overprotective behaviors in the context of child development and health. As a patient-reported outcome (PRO) tool, it captures caregivers’ own perceptions of their protective behaviors [11, 12]. Although general parenting or family functioning measures exist [3, 13, 14], relatively few instruments specifically assess parent overprotection from the caregiver’s perspective. The PPS has been applied in studies involving children with chronic illness, in which higher levels of parental overprotection were associated with perceived child vulnerability and parenting stress [15–17]. However, it has not yet been adapted or validated for use with Korean caregivers.

In Korea, caregiving practices are shaped by a unique cultural context that emphasizes close family ties and strong parental involvement. A strong sense of parental responsibility for child outcomes and heightened concern for children’s safety may further influence protective behaviors [13, 18–22]. This close parental involvement is often viewed as an expression of care and commitment to children’s well-being and success in Korea [18, 22, 23]. However, previous studies have suggested that excessive parental involvement and control may hinder the development of autonomy and independent functioning and may be associated with lower self-directedness, psychological adjustment, and well-being among youth [24–26]. These sociocultural characteristics may affect how parental overprotection is expressed and perceived, particularly among caregivers of children with chronic or rare diseases.

Therefore, this study aimed to evaluate the reliability and construct validity of the translated Korean version of the PPS using exploratory factor analysis (EFA) based on a prespecified four-factor model. A culturally adapted and validated Korean version of the PPS will enable clinicians and researchers to accurately assess protective parental behaviors, supporting culturally sensitive, family-centered care and intervention.

## Methods

### Development of the Korean version of the parent protection scale

The original PPS (Supplementary Table S1) comprises 25 items in four subscales: supervision, separation problems, dependence, and control [4, 10]. It includes seven items each for supervision and separation problems, five items for dependence, and nine items for control. Notably, three items were assigned to multiple subscales: “I urge my child to try new things” and “I allow my child to do things on his or her own” were included in both the separation problems and dependence subscales, while “I blame myself when my child gets hurt” was included in both the separation problems and control subscales. Seven items were reverse-coded. All the items were rated on a 4-point Likert scale ranging from 0 (“never”) to 3 (“always”). The total PPS score was calculated by summing the scores of all 25 items, yielding a possible total score ranging from 0 to 75, with higher scores indicating greater protective parental behavior.

The forward–backward translation procedure was used to translate the original version of PPS (Supplementary Table S1) from English into Korean. Two bilingual general practitioners with formal training in family medicine and preventive medicine independently translated the English questionnaire into Korean. The translated version was then back-translated into English by two bilingual experts with doctoral-level training in preventive medicine. Korean grammar and wording were reviewed by researchers from the National Institute of the Korean Language.

Following the development of the provisional Korean version, cognitive debriefing interviews were conducted to evaluate the clarity, comprehensibility, and cultural appropriateness of the translated items. A total of 29 caregivers participated in the linguistic validation process. Individual interviews lasting approximately 30–40 min were conducted to identify items that were perceived as complex, difficult to understand, uncomfortable, or potentially offensive. Participants were also asked whether there were any additional important concepts that should be included. On the basis of the feedback obtained, all items were retained for subsequent psychometric validation and minor revisions were made to the Korean version to improve clarity and acceptability. The final Korean version was established with the agreement of all the authors (Supplementary Table S2). In the Korean version, the response scale was adapted to range from 1 (“never”) to 4 (“always”), with total scores ranging from 25 to 100, consistent with commonly used formats in Korean psychological measures.

### Study participants and data collection

Caregivers of pediatric patients aged 5 to 10 years who were diagnosed with cancer or rare diseases were recruited from eight hospitals (Supplementary Table S3) between August 1, 2023, and December 31, 2024. Recruitment was conducted through promotional posters and QR codes in relevant hospital departments, allowing potential participants to voluntarily access the study.

After reviewing the study information provided via mobile application and promotional materials, only those who provided electronic informed consent were eligible to participate. Caregivers completed the Korean version of the PPS via a mobile application. A total of 179 caregivers completed the baseline survey between August 11, 2023, and December 24, 2024.

### Ethics approval and consent to participate

This study was approved by the Institutional Review Board (IRB) of Seoul National University Hospital (IRB No. H-2211-077-1378) and was conducted under the ethical principles of the Declaration of Helsinki. Informed consent was obtained via electronic signatures from all participants before enrollment.

### Statistical analysis

#### Participant characteristics

The baseline characteristics of the study participants were summarized using descriptive statistics. Continuous variables were presented as means and standard deviations (SDs), whereas categorical variables were presented as frequencies and percentages. Caregiver characteristics included age (years), sex (male or female), educational level (college graduate or higher vs. high school graduate or below), monthly income (< 3 million KRW vs. ≥3 million KRW), area of residence (capital region, metropolitan city, or other regions), household type (single-parent or two-parent), and primary caregiver status (whether the respondent was the primary caregiver; yes or no). Monthly household income was categorized based on the 2023 minimum cost of living for a standard four-person household in Korea (2,654,743 KRW) [27]. Area of residence was categorized as capital region (Seoul, Gyeonggi, and Incheon), metropolitan city (Busan, Daegu, Gwangju, Daejeon, and Ulsan), or other regions (Gangwon, Chungbuk, Chungnam, Sejong, Jeonbuk, Jeonnam, Gyeongbuk, Gyeongnam, and Jeju). Child characteristics included age (years), sex (male or female), and disease type (pediatric cancers or rare pediatric diseases). Total PPS scores were summarized using the mean and SD. Differences in caregiver and child characteristics according to recruitment site were examined using Fisher’s exact test for categorical variable and Welch’s two-sample t-test for continuous variables.

#### Exploratory factor analysis

All reverse-coded items were rescored before analysis. To assess the adequacy of the data for factor analysis, the Kaiser–Meyer–Olkin (KMO) measure and Bartlett’s test of sphericity were performed. A KMO value > 0.60 and a significant Bartlett’s test (*p* < 0.05) were considered acceptable for factor analysis.

EFA was conducted on all 25 items of the Korean PPS using principal axis factoring with direct oblimin rotation because of the conceptual overlap between the subscales in the original version. The number of factors to extract was determined by examining the scree plot and applying the Kaiser criterion (eigenvalues > 1), thereby enhancing the robustness of factor retention decisions.

The factor structure was evaluated by examining factor loadings (≥ 0.30) and item communalities [28, 29]. Cross-loadings were defined as items with factor loadings ≥ 0.30 on more than one factor [29, 30]. In addition, a difference of less than 0.20 between primary and secondary loadings was considered indicative of substantial cross-loading [30]. The four-factor solution was further evaluated on the basis of cumulative variance explained and factor interpretability. Because direct oblimin rotation was used, the factor correlation matrix was also reviewed.

Convergent validity was assessed by calculating the average variance extracted (AVE) and composite reliability (CR) for each factor based on factor loadings. An AVE value of ≥ 0.50 and a CR value of ≥ 0.70 were considered acceptable [31].

#### Reliability

Internal consistency was evaluated using Cronbach’s alpha for each factor and the total scale. A Cronbach’s alpha of ≥ 0.70 was considered acceptable.

#### Concurrent validity

Concurrent validity was assessed using Pearson correlation coefficients between the sum scores of the PPS subscales and those of the Hospital Anxiety and Depression Scale (HADS-A for anxiety, HADS-D for depression) [32] and the Medical Outcomes Study Social Support Survey (MOS-SSS) [33]. Overprotective parenting has been associated with increased parental anxiety and depression, as well as lower perceived social support [34, 35]; therefore, these measures were selected to evaluate the concurrent validity of the PPS. Correlations were interpreted based on theoretical expectations that overprotective parenting would be associated with greater psychological distress and lower perceived social support [34, 35].

All analyses were conducted using IBM SPSS Statistics version 29 and R version 4.4.2, with a two-tailed significance threshold of *p* < 0.05.

## Results

### Baseline characteristics of the study participants

Among the 179 caregivers who participated in the study, the mean age was 40.4 ± 4.4 years. The majority were female (*n* = 164, 91.6%), college graduates or higher (150, 83.8%), and had a monthly household income of ≥ 3 million KRW (161, 89.9%). Most participants resided in the capital area (108, 60.3%), lived in two-parent households (172, 96.1%), and identified as the child’s primary caregiver (177, 98.9%). The average age of the children was 7.5 **±** 1.8 years, and the majority were male (96, 53.6%) and had rare pediatric diseases (138, 77.1%) (Table 1). No significant differences were observed according to recruitment site (Supplementary Table S4). The mean total PPS score was 56.5 ± 7.8 (Supplementary Fig. 1), suggesting a moderate level of parental overprotective tendencies.

**Table 1:** Baseline characteristics of caregivers and their children

Characteristic	*N* (%)	%
**Total**	179	
**Caregiver characteristics**		
**Age (years)**,** mean ± SD**	40.4 **±** 4.4	
**Sex**		
Male	15	8.4
Female	164	91.6
**Education level**		
College graduate or higher	150	83.8
High school graduate or below	29	16.2
Monthly income (KRW)*		
< 3 million	18	10.1
≥ 3 million	161	89.9
**Area of residence**†		
Capital region	108	60.3
Metropolitan city	21	11.7
Others	50	27.9
**Household type**		
Single-parent	7	3.9
Two-parent	172	96.1
**Primary caregiver status**		
Yes	177	98.9
No	2	1.1
**Child characteristics**		
**Age (years)**,** mean ± SD**	7.5 **±** 1.8	
**Sex**		
Male	96	53.6
Female	83	46.4
**Disease type**		
Pediatric cancers	41	22.9
Rare Pediatric diseases	138	77.1

SD: Standard deviation; KRW: Korean Won

*Categorized based on the minimum cost (2,654,743 KRW) of living for a standard household in 2023

†Capital: Seoul, Gyeonggi, Incheon; metropolitan city: Busan, Daegu, Gwangju, Daejeon, Ulsan; others: Gangwon, Chungbuk, Chungnam, Sejong, Jeonbuk, Jeonnam, Gyeongbuk, Gyeongnam, Jeju

### Exploratory factor analysis

Before the EFA was conducted, the KMO measure of sampling adequacy was acceptable at 0.77. Bartlett’s test of sphericity showed significance (χ²(300) = 1245.48, *p* < 0.001), indicating that the data were suitable for factor analysis.

The scree plot (Supplementary Fig. 2) suggested a four-factor solution based on the elbow point. EFA using principal axis factoring with direct oblimin rotation yielded four interpretable factors, accounting for a total of 44.85% of the cumulative variance (Table 2). Item communalities ranged from 0.23 to 0.60, indicating mostly acceptable levels of shared variance across items. Three items (Nos. 1, 15, and 23) had communalities below 0.30; however, they were retained, as their values exceeded 0.20 (Table 2). Although these values are relatively low, communalities in the range of 0.20–0.30 may be acceptable in exploratory factor analysis, particularly when items are theoretically important and contribute to construct coverage [28]. The seven reverse-coded items also demonstrated acceptable factor loadings and communalities (Table 2) and were retained in the final factor solution.

**Table 2:** Factor loadings, communalities, and convergent validity based on caregiver responses for children aged 5–10 years

Item		Factor				Communality
		1	2	3	4	
1	I blame myself when my child gets hurt.			0.43		0.24
2	I comfort my child immediately when he/she cries.		0.68			0.56
3	I encourage my child to depend on me.*		0.32	0.49		0.43
4	I have difficulty separating from my child.			0.62		0.58
5	I trust my child on his/her own. †			0.45		0.51
6	I let my child make his/her own decisions. *†	0.31		0.45		0.57
7	I have difficulty leaving my child with a babysitter.			0.65		0.48
8	I decide when my child eats.	0.68				0.45
9	I use baby words when I talk to my child. *	0.42			-0.34	0.51
10	I urge my child to try new things. *†		-0.36	0.54		0.53
11	I determine who my child will play with.	0.50				0.32
12	I keep a close watch on my child.		0.68			0.55
13	I feed my child even if he/she can do it alone.				-0.76	0.54
14	I feel comfortable leaving my child with other people.†			0.61		0.37
15	I protect my child from criticism.		0.44			0.29
16	I let my child choose what he/she wears.†				-0.51	0.43
17	I make my child go to sleep at a set time. *	0.37			0.44	0.37
18	I go to my child if he/she cries during the night.		0.65			0.47
19	I encourage my child to play with other children.†		-0.45			0.35
20	I give my child extra attention when he/she clings to me.		0.74			0.56
21	I decide what my child eats.	0.72				0.52
22	I dress my child even if he/she can do it alone.				-0.75	0.60
23	I decide when my child goes to the bathroom. *	0.32			-0.33	0.28
24	I know exactly what my child is doing.		0.47			0.32
25	I allow my child to do things on his/her own. *†			0.31	-0.44	0.40
Eigenvalue		4.82	3.19	1.68	1.53	
% of variance		19.27	12.74	6.71	6.13	
Cumulative %		19.27	32.01	38.72	44.85	
Average variance extracted		0.25	0.31	0.27	0.29	
Composite reliability		0.68	0.79	0.76	0.72	

*Include in two different subscales

†Reverse-scored items

The CR values ranged from 0.68 to 0.79, indicating acceptable internal consistency for most factors. However, the AVE values ranged from 0.25 to 0.31, falling below the recommended threshold of 0.50 and suggesting limited convergent validity. This discrepancy between CR and AVE warrants careful interpretation of the factor-level validity results.

In addition to the EFA results, four conceptual subscales were established on the basis of item content and composition (Supplementary Table S5): Factor 1 (strict behavioral control) included 7 items reflecting structured and controlling parenting over routines and decisions, often limiting child autonomy; Factor 2 (emotional intrusiveness) included 9 items indicating emotionally overinvolved parenting that blurs parent–child emotional boundaries; Factor 3 (anxious ambivalence) consisted of 9 items representing conflicted parenting marked by anxiety and inconsistent support for autonomy; and Factor 4 (autonomy restriction) had 7 items describing unnecessary interference in the child’s self-sufficient behaviors and choices.

Seven items showed cross-loadings (Table 2). Although item removal was considered, these items were retained because their primary loadings were higher than their secondary loadings and they contributed to the conceptual interpretation of the factors, thereby preserving content validity. The cross-loadings may reflect conceptual overlap among constructs or potential ambiguity in translation [29]. The factor correlation matrix (Supplementary Table S6) revealed low to moderate correlations among the factors (*r*= -0.24 to 0.16), supporting the use of an oblique rotation.

### Reliability

Internal consistency was acceptable for the total PPS (α = 0.79). Subscale reliability varied, with Factor 3 (anxious ambivalence; α = 0.75) showing the highest reliability, followed by Factor 4 (autonomy restriction; α = 0.66), Factor 1 (strict behavioral control; α = 0.64), and Factor 2 (emotional intrusiveness; α = 0.57) (Table 3).

**Table 3:** Reliability of subscales based on caregiver responses for children aged 5–10 years

Subscale	No. of items	Cronbach’s α
Factor 1 (Strict behavioral control)	7	0.64
Factor 2 (Emotional intrusiveness)	9	0.57
Factor 3 (Anxious ambivalence)	9	0.75
Factor 4 (Autonomy restriction)	7	0.66
Total PPS	25	0.79

No.: The number of; PPS: Parent Protection Scale

### Concurrent validity

Concurrent validity analyses revealed that Factor 1 (strict behavioral control) and Factor 3 (anxious ambivalence) were significantly negatively correlated with anxiety (HADS-A; *r* = − 0.24, *p* < 0.01 and *r* = − 0.42, *p* < 0.001, respectively), depression (HADS-D; *r* = − 0.21, *p* < 0.01 and *r* = − 0.27, *p* < 0.001), and all subdomains of social support (MOS-SSS; *r* = − 0.29 to − 0.36, all *p* < 0.001). Factor 4 (autonomy restriction) was also significantly negatively associated with anxiety (*r* = − 0.25; *p* < 0.001), depression (*r* = − 0.18; *p* < 0.05), and all social support domains (*r* = − 0.28 to − 0.33; all *p* < 0.001). In contrast, Factor 2 (emotional intrusiveness) was not significantly correlated with any of the external subscales (Table 4).

**Table 4:** Concurrent validity of caregiver responses with HADS and MOS-SSS (ages 5–10)

	Factor 1 (Strict behavioral control)	Factor 2 (Emotional intrusiveness)	Factor 3 (Anxious ambivalence)	Factor 4 (Autonomy restriction)
HADS-A(Anxiety)	-0.24**	-0.05	-0.42***	-0.25***
HADS-D (Depression)	-0.21**	-0.10	-0.27***	-0.18*
MOS-SSS (Emotional/informational support)	-0.30***	0.05	-0.35***	-0.32***
MOS-SSS (Tangible/instrumental support)	-0.31***	0.05	-0.29***	-0.28***
MOS-SSS (Affectionate support)	-0.32***	0.00	-0.36***	-0.32***
MOS-SSS (Positive social interaction)	-0.31***	-0.01	-0.36***	-0.33***

*: *p* < 0.05; **: *p* < 0.01; ***: *p* < 0.001

HADS: Hospital Anxiety and Depression Scale; MOS-SSS: Medical Outcomes Study Social Support Survey

## Discussion

This study evaluated the reliability and validity of the Korean version of the PPS among caregivers of children aged 5 to 10 years with cancer or a rare disease. The EFA identified a four-factor structure—strict behavioral control, emotional intrusiveness, anxious ambivalence, and autonomy restriction—that partially aligned with the original PPS while also showing differences in item composition and organization (Supplementary Table S7).

Although the factor labels differed from those of the original PPS (supervision, separation problems, dependence, and control), their conceptual contents broadly overlapped. Strict behavior control and autonomy restriction reflected behavioral supervision and control, whereas emotional intrusiveness and anxious ambivalence represented emotional and relational aspects embedded within separation and dependence domains. Compared with the original PPS, which emphasized behavioral aspects [3, 4], the Korean version captured both behavioral and emotional dimensions of parental protection, suggesting that caregivers may interpret protective behaviors not only in terms of behavioral monitoring but also in relation to emotional involvement and responsiveness [13, 24, 36]. Protective parenting in Korean family contexts may be expressed not only through behavioral monitoring and supervision but also through emotional involvement and relational closeness. Previous studies have suggested that parental commitment and concern are often communicated through intensive involvement in children’s daily lives [21, 22, 37], making emotional and behavioral aspects of protection more closely intertwined than suggested by the original PPS framework.

Notably, several items were reclassified or showed cross-loadings. These patterns were based on empirical criteria, including factor loadings, cross-loadings, and differences between primary and secondary loadings, as well as interpretation of item content, as described in the Results section. For example, autonomy-related items were regrouped under anxious ambivalence or autonomy restriction. These patterns may partly reflect cultural meanings attached to parental involvement and autonomy in Korean family contexts. Previous studies have suggested that close parental involvement and guidance are often viewed as expressions of parental responsibility, care, and commitment to children’s well-being [18, 21, 22]. Within this context, autonomy-related behaviors may be interpreted not simply in terms of promoting independence, but also in relation to maintaining parental involvement and ensuring children’s safety. Such interpretations may be particularly salient among caregivers of children with chronic illness, for whom fostering autonomy is often balanced against concerns regarding health management and perceived vulnerability [15, 16]. Consequently, autonomy-related items may have been more closely associated with emotional concern and behavioral restriction, contributing to their loading on the anxious ambivalence and autonomy restriction factors.

Convergent validity showed mixed results, with acceptable CR but low AVE values, suggesting that although internal consistency was generally adequate, the constructs may not be fully captured by the current item set. Similarly, although the PPS demonstrated acceptable internal consistency at the total scale level, subscale reliability was variable. These findings may reflect the exploratory nature of the factor structure, overlap among constructs, heterogeneity in caregiving experiences, and differences in how respondents interpret specific items, highlighting the need for further validation and refinement of the scale in future studies [28, 29].

The emotional intrusiveness factor showed relatively lower reliability under 0.60. This may indicate that emotional involvement is interpreted variably depending on caregiving context, particularly when it reflects concern for the child’s health or future outcomes [18, 23]. As a result, such behaviors may not be consistently distinguished as a separate construct by caregivers in this population.

Concurrent validity analyses revealed that higher levels of strict behavioral control, anxious ambivalence, and autonomy restriction were associated with lower caregiver anxiety, depression, and perceived social support. Although previous studies have often reported that overprotective parenting is associated with greater psychological distress [2, 15–17], most of these studies were also conducted among caregivers of children with chronic illness or cancer in Western settings. Therefore, the inverse associations observed in this study may nor be explained solely illness context. Cultural difference in the meaning and function of parenting behaviors may also be relevant [13]. In Korean family contexts, protective parenting may be perceived as an enactment of parental responsibility and commitment rather than primarily as a source of relational strain or psychological burden [18, 21–23]. Consequently, greater behavioral monitoring and control may help caregivers manage illness-related uncertainty and feel more confident in responding to their child’s care needs [38]. Future longitudinal studies are needed to determine whether protective parenting functions as an adaptive coping strategy that mitigates caregiver distress or whether lower distress enables greater engagement in protective parenting behaviors.

Consistent with these findings, although no established clinical cutoffs are available for the PPS, the distribution of scores in this study suggests variability in protective parenting behaviors rather than uniformly elevated overprotection. This pattern may reflect differences in how caregivers adapt their behaviors to caregiving demands.

Caregiving in the context of serious pediatric illness is characterized by ongoing uncertainty and heightened perceptions of child vulnerability [16, 37]. Under these conditions, protective behaviors may reflect adaptive responses to complex caregiving demands rather than maladaptive overprotection [5, 6]. Caregivers often experience increased emotional and practical burdens because of the ongoing management of complex health needs, which may intensify their protective responses and affect the balance between fostering independence and ensuring safety [5, 6]. This perspective is consistent with previous findings that caregiving involves balancing safety, uncertainty, and developmental needs [2], and highlights the importance of interpreting protective behaviors within their clinical context.

Anxious ambivalence, a newly identified factor, may capture the tension between promoting independence and ensuring safety. This dynamic is particularly relevant for caregivers of children with serious health conditions, who must support autonomy while managing risk [5, 6]. In family-oriented cultural contexts such as Korea, this balance may be further shaped by expectations of close parental involvement and responsibility for child outcomes [21, 22]. Furthermore, strict behavioral control and autonomy restriction may represent forms of parenting that are not necessarily perceived as problematic in this context. In situations involving chronic illness, structured and closely involved parenting may be viewed as necessary and appropriate rather than excessive. Previous research suggests that parenting behaviors emphasizing control and involvement may be interpreted differently depending on cultural and contextual factors [13, 21, 22], highlighting the need to distinguish between adaptive and maladaptive forms of protection.

This study has several strengths. First, the use of rigorous backward translation procedures and comprehensive EFA allowed culturally sensitive adaptation and revealed meaningful differences in the factor structure compared with the original Western version. Second, multi-institutional recruitment from eight hospitals nationwide enhanced the specificity of the findings within this clinical population, which is consistent with the original PPS study. These insights contribute to a deeper understanding of protective parental behaviors in the Korean cultural context, particularly among caregivers facing chronic pediatric illnesses.

However, several limitations should be noted. First, the current analysis utilizes only cross-sectional baseline data. Therefore, the ability to assess predictive validity or test–retest reliability is limited. Second, the current study participants were homogeneous in terms of age, sex, educational level, and income, with an overrepresentation of mothers and highly educated, high socioeconomic status households. More than half of the households reported monthly incomes above the national average for Korean families, which may further restrict their applicability to lower-income groups. Although some studies suggest that overprotective parenting is more prevalent and impactful in middle- to high-income families [25, 39], other studies report no significant association between socioeconomic status and overprotection [19, 40]. Therefore, future studies should include more demographically diverse samples to better understand protective parenting across different social strata. Third, three of the subscales (strict behavioral control, emotional intrusiveness, and autonomy restriction) showed questionable internal consistency (α < 0.70), indicating the need for further psychometric refinement or the development of new culturally sensitive items. This also suggests a cautious interpretation of related findings. Fourth, three items with low communalities (below 0.30; ranging from 0.24 to 0.29) were retained, indicating weak associations with the extracted factors and suggesting the need for future item revision or removal. In addition, seven items showed cross-loadings on two different subscales, with minimal differences in factor loadings, raising concerns about discriminant validity. These results may have been influenced by the relatively small sample size (*N* = 179), which can lead to an unstable factor structure, reduced reproducibility, limited generalizability, and decreased statistical power.

Despite these limitations, the Korean version of the PPS offers preliminary evidence of cultural relevance and psychometric utility. The adapted scale provides a useful tool for clinicians and researchers aiming to assess protective parenting behaviors in Korea and supports the broader goal of culturally sensitive, family-centered care in pediatric settings [2, 5, 6]. Continued refinement of such culturally adapted measures is essential for improving targeted interventions and advancing cross-cultural parenting research [13, 24]. Furthermore, as this is the first study to validate the Korean version of the PPS among caregivers of children with serious illnesses, further research is necessary to extend its validation. For example, exploring how cultural differences in relational sensitivity influence PPS responses would help distinguish culturally specific parenting expectations from underlying psychological constructs such as parental mentalization. This approach could broaden the understanding of parental behaviors across diverse contexts and illuminate the complex interplay between culture, parental representations of their children, and caregiver self-reflection.

## Conclusion

This study provides preliminary evidence supporting the use of the Korean version of the PPS as a culturally sensitive and reliable measure of parental protection. The adapted scale effectively captures culturally specific aspects of protective parental behaviors and has meaningful associations with parental psychological well-being. These findings highlight the potential utility of the Korean PPS in both research and clinical settings. However, further psychometric refinement, expanded validation across diverse populations, and continued development of culturally sensitive assessment tools are necessary to enhance its robustness and applicability. Overall, the Korean PPS represents a valuable contribution to understanding and supporting parenting practices within the Korean cultural context. In clinical practice, the Korean PPS may provide a structured approach to identifying specific dimensions of protective parenting, enabling clinicians to assess caregiving patterns more precisely and to develop targeted psychoeducational or supportive interventions that promote an appropriate balance between protection and child autonomy.

## Supplementary Information

Below is the link to the electronic supplementary material.

Supplementary Material 1

## Data Availability

The dataset contains personal information and is not publicly available. It may be shared upon reasonable request with participants’ explicit consent.

## Abbreviations

EFA: Exploratory factor analysis
SD: Standard deviation
AVE: Average Variance Extracted
CR: Composite Reliability
HADS: Hospital Anxiety and Depression Scale
HADS-A: HADS for Anxiety
HADS-D: HADS for Depression
IRB: Institutional Review Board
KMO: Kaiser–Meyer–Olkin
MOS-SSS: Medical Outcomes Study Social Support Survey
PPS: Parent Protection Scale
PRO: Patient-reported Outcome

## References

[1] Bowlby J (1988) A secure base: Parent-child attachment and healthy human development. A secure base: Parent-child attachment and healthy human development. New York, NY, US: Basic Books. xii, 205–xii, 205

[2] Young B et al (2002) Parenting in a crisis: conceptualising mothers of children with cancer. Soc Sci Med 55(10):1835–1847 10.1016/s0277-9536(01)00318-5 12383468

[3] Thomasgard M, Metz WP (1997) Parental overprotection and its relation to perceived child vulnerability. Am J Orthopsychiatry 67(2):330–335 10.1037/h0080237 9142367

[4] Thomasgard M et al (1995) Parent-child relationship disorders. Part I. Parental overprotection and the development of the Parent Protection Scale. J Dev Behav Pediatr 16(4):244–250 7593659

[5] Knafl KA, Gilliss CL (2002) Families and Chronic Illness: A Synthesis of Current Research. J Fam Nurs 8(3):178–198

[6] Pelentsov LJ, Fielder AL, Esterman AJ (2016) The Supportive Care Needs of Parents With a Child With a Rare Disease: A Qualitative Descriptive Study. J Pediatr Nurs 31(3):e207–e218 10.1016/j.pedn.2015.10.022 26651231

[7] Levy DM (1966) Maternal overprotection. New York: Norton, New York

[8] Roe A, Siegelman M (1963) A parent-child relations questionnaire. Child Dev 34:355–369 10.1111/j.1467-8624.1963.tb05143.x 13982326

[9] Parker G, Tupling H, Brown LB (1979) A Parental Bonding Instrument. Br J Med Psychol 52(1):1–10

[10] Thomasgard M, Metz WP (1993) Parental overprotection revisited. Child Psychiatry Hum Dev 24(2):67–80 10.1007/BF02367260 8287694

[11] Weldring T, Smith SM (2013) Patient-Reported Outcomes (PROs) and Patient-Reported Outcome Measures (PROMs). Health Serv Insights 6:61–68 10.4137/HSI.S11093 PMC4089835 25114561

[12] Deshpande PR et al (2011) Patient-reported outcomes: A new era in clinical research. Perspect Clin Res 2(4):137–144 10.4103/2229-3485.86879 PMC3227331 22145124

[13] Bornstein MH (2012) Cultural Approaches to Parenting. Parent Sci Pract 12(2–3):212–221 10.1080/15295192.2012.683359 PMC3433059 22962544

[14] Park J et al (2015) Standardization Study for the Korean Version of Parenting Relationship Questionnaire-Children. Hum Ecol Res 53(2):169–177

[15] Hullmann SE et al (2010) Parental overprotection, perceived child vulnerability, and parenting stress: a cross-illness comparison. J Clin Psychol Med Settings 17(4):357–365 10.1007/s10880-010-9213-4 21086027

[16] Mullins LL et al (2007) The relationship of parental overprotection, perceived child vulnerability, and parenting stress to uncertainty in youth with chronic illness. J Pediatr Psychol 32(8):973–982 10.1093/jpepsy/jsm044 17634187

[17] Tillery R, Long A, Phipps S (2014) Child perceptions of parental care and overprotection in children with cancer and healthy children. J Clin Psychol Med Settings 21(2):165–172 10.1007/s10880-014-9392-5 PMC4032611 24682801

[18] Kwon K-A, Yoo G, De Gagne JC (2017) Does Culture Matter? A Qualitative Inquiry of Helicopter Parenting in Korean American College Students. J Child Fam stud 26(7):1979–1990

[19] Chung YS, Park KJ (2021) Development and Validation of a Maternal Overprotection Scale for Young Children. Korean J Child Stud 42(3):359–370

[20] Lee HY, Park JH, Kim TW (2021) Cross-cultural adaptation and psychometric validation of the Korean version of rehabilitation complexity scale for the measurement of complex rehabilitation needs. Med (Baltim) 100(24):e26259 10.1097/MD.0000000000026259 PMC8213332 34128854

[21] Kim E, Hong S (2007) First-generation Korean-American parents’ perceptions of discipline. J Prof Nurs 23(1):60–68 10.1016/j.profnurs.2006.12.002 PMC1850109 17292135

[22] Park YS et al (2010) Acculturation, enculturation, parental adherence to Asian cultural values, parenting styles, and family conflict among Asian American college students. Asian Am J Psychol 1(1):67–79

[23] Lee HN et al (2024) Acculturation, Enculturation, Culture-Specific Emotional Distress, and Parenting Among Korean Immigrant Mothers. Asian Am J Psychol 15(2):73–82 10.1037/aap0000328 PMC11343448 39184346

[24] Wang Q, Pomerantz EM, Chen H (2007) The role of parents’ control in early adolescents’ psychological functioning: a longitudinal investigation in the United States and China. Child Dev 78(5):1592–1610 10.1111/j.1467-8624.2007.01085.x 17883450

[25] Chung IJ (2012) The influences of parental neglect, overprotection and private tutoring on development in early childhood. Korean J Early Child Educ 32(4):255–278

[26] Choi A, Lee S (2019) What Makes Korean Youth Happy: Self-Directedness Mediates the Relationship between Parenting and Subjective Well-Being. Hum Ecol Res 57(4):499–512

[27] *Minimum cost of living for a standard household of four*. [cited (2025) July 26]; Available from: https://kosis.kr/statHtml/statHtml.do?sso=ok&returnurl=https%3A%2F%2Fkosis.kr%3A443%2FstatHtml%2FstatHtml.do%3Flist_id%3DG_5%26obj_var_id%3D%26seqNo%3D%26query%3D%ED%91%9C%EC%A4%80%EA%B0%80%EA%B5%AC%EC%9D%98+%EC%B5%9C%EC%A0%80%EC%83%9D%EA%B3%84%EB%B9%84%26tblId%3DDT_117097_N001%26vw_cd%3DMT_ZTITLE%26language%3Dkor%26orgId%3D117%26conn_path%3DMT_ZTITLE%26itm_id%3D%26lang_mode%3Dko%26

[28] MacCallum RC et al (2001) Sample Size in Factor Analysis: The Role of Model Error. Multivar Behav Res 36(4):611–637 10.1207/S15327906MBR3604_06 26822184

[29] Worthington RL, Whittaker TA (2006) Scale Development Research:A Content Analysis and Recommendations for Best Practices. Couns Psychol 34(6):806–838

[30] Hair JF et al (2009) Multivariate Data Analysis, 7 edn. Pearson Prentice Hall, Upper Saddle River, NJ

[31] Fornell C, Larcker DF (1981) Evaluating Structural Equation Models with Unobservable Variables and Measurement Error. J Mark Res 18(1):39–50

[32] Zigmond AS, Snaith RP (1983) The Hospital Anxiety and Depression Scale. Acta psychiatrica Scandinavica 67(6):361–370 10.1111/j.1600-0447.1983.tb09716.x 6880820

[33] Sherbourne CD, Stewart AL (1991) The MOS social support survey. Soc Sci Med 32(6):705–714 10.1016/0277-9536(91)90150-b 2035047

[34] Schiffrin HH et al (2014) Helping or Hovering? The Effects of Helicopter Parenting on College Students’ Well-Being. J Child Fam stud 23(3):548–557

[35] Segrin C et al (2012) The Association Between Overparenting, Parent-Child Communication, and Entitlement and Adaptive Traits in Adult Children. Fam Relat 61(2):237–252

[36] Barber BK (1996) Parental psychological control: revisiting a neglected construct. Child Dev 67(6):3296–3319 9071782

[37] Domaradzki J, Jabkowski P, Walkowiak D (2025) Rare disease, common struggles: quality of life, caregiver burden and financial wellbeing of family caregivers in Poland. Sci Rep 15(1):22678 10.1038/s41598-025-08866-7 PMC12217616 40595391

[38] Mishel MH (1988) Uncertainty in illness. Image J Nurs Sch 20(4):225–232 10.1111/j.1547-5069.1988.tb00082.x 3203947

[39] Bernstein G, Triger ZH (2011) Over-Parenting. UC Davis Law Review, 44(4): p. 1221

[40] Gagnon RJ (2019) Examining overparenting, socioeconomic status, and parental out-of-school time experience: Does socioeconomic status and out-of-school-time experience matter? Child Youth Serv Rev 101:181–189

