# Supplementary material for: Validation study of the Korean version of the parent protection scale
Source: J Patient Rep Outcomes. 2026 Aug 5;10:199. doi: 10.1186/s41687-026-01170-6 (PMC13737214; doi:10.1186/s41687-026-01170-6)
Supplement: Supplementary file 1 [file 41687_2026_1170_MOESM1_ESM.docx]

**Supplementary Materials**

**Tables**

**Supplementary Table S1.** Original items of the Parent Protection Scale.

**Supplementary Table S2.** Translated items of the Korean version of the Parent Protection Scale.

**Supplementary Table S3.** Hospitals and the number of caregiver participants who participated in the study.

**Supplementary Table S4.** Comparison of caregiver and child characteristics by recruitment site group.

**Supplementary Table S5.** Subscale grouping and rationales for the Korean Parent Protection Scale.

**Supplementary Table S6.** Factor correlation matrix based on caregiver responses for children aged 5–10 years.

**Supplementary Table S7.** Comparison of original and Korean versions of the Parent Protection Scale by subscales and items.

**Figures**

**Supplementary Figure 1.** Distribution of total Parent Protection Scale scores.

**Supplementary Figure 2.** Scree plot for determining the number of factors based on the inflection point.

**Supplementary Table S1.** Original items of the Parent Protection Scale.

| **No.** | **Original items in English** | **Supervision** | | **Separation Problems** | | **Dependence** | **Control** | |
| --- | --- | --- | --- | --- | --- | --- | --- | --- |
| 1 | I blame myself when my child gets hurt.* |  | | ■ | |  | ■ | |
| 2 | I comfort my child immediately when he/she cries. | ■ | |  | |  |  | |
| 3 | I encourage my child to depend on me. | ■ | |  | |  |  | |
| 4 | I have difficulty separating from my child. |  | | ■ | |  |  | |
| 5 | I trust my child on his/her own.† |  | |  | | ■ |  | |
| 6 | I let my child make his/her own decisions.† |  | |  | | ■ |  | |
| 7 | I have difficulty leaving my child with a babysitter. |  | | ■ | |  |  | |
| 8 | I decide when my child eats. |  | |  | |  | ■ | |
| 9 | I use baby words when I talk to my child. |  | |  | |  | ■ | |
| 10 | I urge my child to try new things.*† |  | | ■ | | ■ |  | |
| 11 | I determine who my child will play with. |  | |  | |  | ■ | |
| 12 | I keep a close watch on my child. | ■ | |  | |  |  | |
| 13 | I feed my child even if he/she can do it alone. |  | |  | |  | ■ | |
| 14 | I feel comfortable leaving my child with other people.† |  | | ■ | |  |  | |
| 15 | I protect my child from criticism. | ■ | |  | |  |  | |
| 16 | I let my child choose what he/she wears.† |  | |  | | ■ |  | |
| 17 | I make my child go to sleep at a set time. |  | |  | |  | ■ | |
| 18 | I go to my child if he/she cries during the night. | ■ | |  | |  |  | |
| 19 | I encourage my child to play with other children.† |  | | ■ | |  |  | |
| 20 | I give my child extra attention when he/she clings to me. | ■ | |  | |  |  | |
| 21 | I decide what my child eats. |  | |  | |  | ■ | |
| 22 | I dress my child even if he/she can do it alone. |  | |  | |  | ■ | |
| 23 | I decide when my child goes to the bathroom. |  | |  | |  | ■ | |
| 24 | I know exactly what my child is doing. | ■ | |  | |  |  | |
| 25 | I allow my child to do things on his/her own.*† |  | | ■ | | ■ |  | |
| *Include in two different subscales.  †Reverse-scored items. | |  |  | |  | | |  |

**Supplementary Table S2.** Translated items of the Korean version of the Parent Protection Scale.

| **No.** | **Items in Korean** |
| --- | --- |
| 1 | 자녀가 다쳤을 때 부모인 나 자신을 탓한다. |
| 2 | 자녀가 울면 즉시 달래 준다. |
| 3 | 자녀가 나에게 의존하도록 한다. |
| 4 | 자녀와 떨어지기가 어렵다. |
| 5 | 자녀가 혼자일 때 스스로 알아서 잘할 것이라고 믿는다. * |
| 6 | 자녀가 스스로 결정을 내리도록 한다. * |
| 7 | 자녀를 육아도우미 또는 돌봄 선생님에게 맡기기가 어렵다. |
| 8 | 자녀의 식사 시간은 내가 결정한다. |
| 9 | 자녀와 대화할 때 어린 아기를 대하듯이 말한다. |
| 10 | 자녀가 새로운 것을 시도해 보도록 한다. * |
| 11 | 자녀가 누구와 놀지는 내가 결정한다. |
| 12 | 자녀를 주의 깊게 지켜본다. |
| 13 | 자녀가 혼자 밥을 먹을 수 있어도 밥을 먹여 준다. |
| 14 | 자녀를 다른 사람에게 맡길 때 마음이 편하다. * |
| 15 | 자녀를 비판으로부터 보호한다. |
| 16 | 자녀가 자신이 입을 옷을 직접 고르도록 한다. * |
| 17 | 자녀가 정해진 시간에 잘 수 있도록 한다. |
| 18 | 자녀가 밤에 울면 즉시 아이에게로 간다. |
| 19 | 자녀가 다른 아이들과 어울려 놀도록 격려한다. * |
| 20 | 자녀가 나에게 매달릴 때 주의를 기울인다. |
| 21 | 자녀가 무엇을 먹을지는 내가 결정한다. |
| 22 | 자녀가 혼자 옷을 입을 수 있어도 옷을 입혀 준다. |
| 23 | 자녀가 화장실에 가는 시간은 내가 결정한다. |
| 24 | 자녀가 무엇을 하는지 정확히 알고 있다. |
| 25 | 자녀가 할 수 있는 일은 스스로 해 보도록 한다. * |
| *Reverse-scored items | |

**Supplementary Table S3.** Hospitals and the number of caregiver participants who participated in the study.

| **Name** | **Participants (caregivers)** |
| --- | --- |
| Seoul National University Hospital | 158 |
| Seoul National University Bundang Hospital | 11 |
| Pusan National University Yangsan Hospital | 3 |
| Samsung Medical Center | 3 |
| Asan Medical Center | 1 |
| Jeonbuk National University Hospital | 1 |
| National Cancer Center | 1 |
| Samsung Changwon Hospital | 1 |
| **Total** | **179** |

**Supplementary Table S4.** Comparison of caregiver and child characteristics by recruitment site group. (N=179)

| **Characteristic** | **Seoul National**  **University Hospital, n (%)** | **Others, n (%)** | **p-value*** |
| --- | --- | --- | --- |
| **Total** | 158 (88.3) | 21 (11.7) |  |
| **Caregiver characteristics** |  |  |  |
| **Age (years), mean±SD** | 40.4±4.4 | 40.2±4.3 | 0.829 |
| **Sex** |  |  | >0.999 |
| Male | 14 (8.9) | 1 (4.8) |  |
| Female | 144 (91.1) | 20 (95.2) |  |
| **Education level** |  |  | 0.344 |
| College graduate or higher | 134 (84.8) | 16 (76.2) |  |
| High school graduate or below | 24 (15.2) | 5 (23.8) |  |
| **Monthly income (KRW)** † |  |  | 0.699 |
| <3 million | 17 (10.8) | 1 (4.8) |  |
| ≥3 million | 141 (89.2) | 20 (95.2) |  |
| **Area of residence**‡ |  |  | 0.942 |
| Capital region | 94 (59.5) | 14 (66.7) |  |
| Metropolitan city | 19 (12.0) | 2 (9.5) |  |
| Others | 45 (28.5) | 5 (23.8) |  |
| **Household type** |  |  | 0.589 |
| Single-parent | 6 (3.8) | 1 (4.8) |  |
| Two-parent | 152 (96.2) | 20 (95.2) |  |
| **Primary caregiver status** |  |  | >0.999 |
| Yes | 156 (98.7) | 21 (100.0) |  |
| No | 2 (1.3) | 0 (0.0) |  |
| **Child characteristics** |  |  |  |
| **Age (years), mean±SD** | 7.4±1.7 | 8.1±1.8 | 0.103 |
| **Sex** |  |  | 0.354 |
| Male | 87 (55.1) | 9 (42.9) |  |
| Female | 71 (44.9) | 12 (57.1) |  |
| **Disease type** |  |  | 0.168 |
| Pediatric cancers | 39 (24.7) | 2 (9.5) |  |
| Rare Pediatric diseases | 119 (75.3) | 19 (90.5) |  |
| N: the number of corresponding cases; SD: Standard deviation; KRW: Korean Won.  *Calculated using Fisher’s exact test for categorical variables and Welch two-sample t-test for continuous variables.  †Categorized based on the minimum cost (2,654,743 KRW) of living for a standard household in 2023.  ‡Capital: Seoul, Gyeonggi, Incheon; metropolitan city: Busan, Daegu, Gwangju, Daejeon, Ulsan; others: Gangwon, Chungbuk, Chungnam, Sejong, Jeonbuk, Jeonnam, Gyeongbuk, Gyeongnam, Jeju. | | | |

**Supplementary Table S5.** Subscale grouping and rationales for the Korean Parent Protection Scale.

| **Subscale/item (No. of items)** | **Rationale** |
| --- | --- |
| **Factor 1: Strict behavioral control (7)** |  |
| I let my child make his/her own decisions. *† | This factor reflects a highly structured parenting style, where **the parent maintains tight behavioral control over the child’s routines and decisions.** This factor combines authoritative scheduling with subtle infantilization or limiting autonomy, even in tasks children can perform themselves. |
| I decide when my child eats. |  |
| I use baby words when I talk to my child. * |  |
| I determine who my child will play with. |  |
| I make my child go to sleep at a set time. * |  |
| I decide what my child eats. |  |
| I decide when my child goes to the bathroom.* |  |
| **Factor 2: Emotional intrusiveness (9)** |  |
| I comfort my child immediately when he/she cries. | This factor highlights **emotional overinvolvement and vigilance.** Parents in this group may have trouble separating their own emotional responses from the child's, showing intrusive affection and emotional regulation. It reflects **difficulty respecting the child’s emotional boundaries.** |
| I encourage my child to depend on me. * |  |
| I urge my child to try new things. † |  |
| I keep a close watch on my child. |  |
| I protect my child from criticism. |  |
| I encourage my child to play with other children. † |  |
| I go to my child if he/she cries during the night. |  |
| I give my child extra attention when he/she clings to me. |  |
| I know exactly what my child is doing. |  |
| **Factor 3: Anxious ambivalence (9)** |  |
| I blame myself when my child gets hurt. | This factor captures **emotional ambivalence toward child autonomy**, often driven by parental anxiety. These parents **want to support autonomy but feel distressed or guilty** about doing so, resulting in inconsistent or contradictory behaviors. It reflects **psychological conflict about dependence vs. independence.** |
| I encourage my child to depend on me.* |  |
| I have difficulty separating from my child. |  |
| I trust my child on his/her own. † |  |
| I let my child make his/her own decisions. † |  |
| I have difficulty leaving my child with a babysitter. |  |
| I urge my child to try new things. † |  |
| I feel comfortable leaving my child with other people. † |  |
| I allow my child to do things on his/her own. † |  |
| **Factor 4: Autonomy restriction (7)** |  |
| I use baby words when I talk to my child. * | This factor focuses on **limiting the child’s self-sufficiency**, even in developmentally appropriate areas. This factor represents **a lack of trust in the child’s capability**, leading to unnecessary parental intervention in basic self-care and choices. |
| I feed my child even if he/she can do it alone. |  |
| I let my child choose what he/she wears. † |  |
| I make my child go to sleep at a set time. * |  |
| I dress my child even if he/she can do it alone. |  |
| I decide when my child goes to the bathroom. |  |
| I allow my child to do things on his/her own. † |  |
| *Include in two different subscales.  †Reverse-scored items. | |

**Supplementary Table S6.** Factor correlation matrix based on caregiver responses for children aged 5–10 years.

|  | **Factor 1**  **(Strict behavioral control)** | **Factor 2**  **(Emotional intrusiveness)** | **Factor 3**  **(Anxious ambivalence)** | **Factor 4**  **(Autonomy restriction)** |
| --- | --- | --- | --- | --- |
| **Factor 1**  **(Strict behavioral control)** | 1.00 | 0.05 | 0.16 | -0.21 |
| **Factor 2**  **(Emotional intrusiveness)** |  | 1.00 | -0.01 | 0.05 |
| **Factor 3**  **(Anxious ambivalence)** |  |  | 1.00 | -0.24 |
| **Factor 4**  **(Autonomy restriction)** |  |  |  | 1.00 |

**Supplementary Table S7.** Item-level comparison of subscale assignment in the original and Korean versions of the Parent Protection Scale.

| **Item** | **Original subscale** | **Korean subscale** |
| --- | --- | --- |
| I blame myself when my child gets hurt. | Separation problems / Control | Anxious ambivalence |
| I comfort my child immediately when he/she cries. | Supervision | Emotional intrusiveness / Anxious ambivalence |
| I encourage my child to depend on me. | Supervision | Emotional intrusiveness |
| I have difficulty separating from my child. | Separation problems | Anxious ambivalence |
| I trust my child on his/her own. * | Dependence | Anxious ambivalence |
| I let my child make his/her own decisions. * | Dependence | Strict behavioral control / Anxious ambivalence |
| I have difficulty leaving my child with a babysitter. | Separation problems | Anxious ambivalence |
| I decide when my child eats. | Control | Strict behavioral control |
| I use baby words when I talk to my child. | Control | Strict behavioral control / Autonomy restriction |
| I urge my child to try new things. * | Separation problems / Dependence | Emotional intrusiveness / Anxious ambivalence |
| I determine who my child will play with. | Control | Strict behavioral control |
| I keep a close watch on my child. | Supervision | Emotional intrusiveness |
| I feed my child even if he/she can do it alone. | Control | Autonomy restriction |
| I feel comfortable leaving my child with other people. * | Separation problems | Anxious ambivalence |
| I protect my child from criticism. | Supervision | Emotional intrusiveness |
| I let my child choose what he/she wears. * | Dependence | Autonomy restriction |
| I make my child go to sleep at a set time. | Control | Strict behavioral control / Autonomy restriction |
| I go to my child if he/she cries during the night. | Supervision | Emotional intrusiveness |
| I encourage my child to play with other children. * | Separation problems | Emotional intrusiveness |
| I give my child extra attention when he/she clings to me. | Supervision | Emotional intrusiveness |
| I decide what my child eats. | Control | Strict behavioral control |
| I dress my child even if he/she can do it alone. | Control | Autonomy restriction |
| I decide when my child goes to the bathroom. | Control | Strict behavioral control / Autonomy restriction |
| I know exactly what my child is doing. | Supervision | Emotional intrusiveness |
| I allow my child to do things on his/her own. * | Separation problems / Dependence | Anxious ambivalence / Autonomy restriction |
| *Reverse-scored items | | |

**Supplementary Figure 1.** Distribution of total Parent Protection Scale scores.

N: number of participants; SD: standard deviation; Min: minimum; Max: maximum; PPS: Parent Protection Scale.

**Supplementary Figure 2.** Scree plot for determining the number of factors based on the inflection point.
